# Assessing knowledge about lymphatic filariasis and the implementation of mass drug administration amongst drug deliverers in three districts/cities of Indonesia

**DOI:** 10.1186/s13071-018-2881-x

**Published:** 2018-05-25

**Authors:** Christiana R. Titaley, Rita Damayanti, Nugroho Soeharno, Anifatun Mu’asyaroh, Mark Bradley, Tim Lynam, Alison Krentel

**Affiliations:** 1 Center for Health Research Universitas Indonesia. Faculty of Public Health Universitas Indonesia, Depok, West Java Province Indonesia; 2 NTD’s Global Health Programs, GSK, Brentford, UK; 3 Reflecting Society Pty Ltd., Townsville, Queensland Australia; 4 Bruyere Research Institute, Ottawa, Canada; 5 London School of Hygiene and Tropical Medicine, London, UK

**Keywords:** Lymphatic filariasis, Mass drug administration (MDA), Drugs deliverer, Knowledge, Feedback, Indonesia, Community drug distributor

## Abstract

**Background:**

This research assesses knowledge amongst drug deliverers about the implementation of mass drug administration (MDA) for lymphatic filariasis (LF) in Agam District (West Sumatera Province), the City of Depok (West Java Province) and the City of Batam (Kepulauan Riau Province), Indonesia.

**Methods:**

A cross-sectional survey was conducted from January to March 2015 at these three sites. Respondents were identified using purposive sampling (i.e. cadre, health worker or community representatives). A total of 318 questionnaires were accepted for analysis. Three outcomes were assessed: knowledge about LF; knowledge about MDA implementation; and was informed about MDA coverage. Logistic regression analyses were employed to examine factors associated with these three outcomes.

**Results:**

Less than half of respondents were charactersised as having a high level of LF knowledge and less than half a high level of knowledge about MDA. The odds of having a high level of knowledge of LF was significantly lower in Batam City than Agam District, yet higher amongst health workers than cadres. Deliverers living in urban areas reported more feedback on MDA outcomes than in the rural district. Health workers received more feedback than cadres (*P* < 0.001). Deliverers perceived the difference between coverage (drug receipt) and compliance (drug ingestion) in the community.

**Conclusions:**

There are variations in knowledge about LF and MDA as well as feedback across drug deliverers in MDA across geographical areas. Adaptation of the MDA guidelines, supportive supervision, increasing the availability of supporting materials and directly-observed therapy might be beneficial to improve coverage and compliance in all areas.

## Background

Lymphatic filariasis (LF) is one of the oldest parasitic diseases in the world [1]. Although it does not kill, LF disables individuals with its long-term clinical manifestations which can also result in economic and social consequences [2]. To date, more than 120 million people in 81 countries are infected globally and more than one billion people continue to live in areas at risk for infection [1].

In Indonesia, LF remains an important public health problem. Efforts to eliminate LF in Indonesia have been conducted since 1975, especially in highly endemic areas [3]. Indonesia is the only country in the world with three types of LF parasite: *Wuchereria bancrofti*, *Brugia malayi* and *Brugia timori.* It was estimated that in 2016, 29 provinces and 239 cities/districts were LF endemic areas, and thereby 102,279,739 people living in those areas were at risk of infection with LF [4].

In 2000, the Global Programme to Eliminate LF (GPELF) was established by the World Health Organization, with a target of LF elimination by 2020 [1]. GPELF uses a two-pronged approach, combining mass drug administration (MDA) for all those eligible in at risk populations [5] with assistance for those with lymphedema and elephantiasis to reduce LF morbidity and suffering [1]. It is thought that MDA given for long enough, at high enough coverage and adherence (compliance) will be sufficient to interrupt transmission and eliminate the parasite [1, 6, 7]. Districts are deemed eligible for MDA if they have greater than 1% microfilaria prevalence (mf) in the total population [1]. Indonesia has been participating in the global LF elimination programme since April 2002 using a district or city as its implementation unit [8]. Presidential Regulation No. 7 Year 2005 listed the elimination of LF as a national priority for controlling infectious diseases [9].

One of the key components in the Indonesian MDA programme is the use of community drug distributors, called cadres in Indonesia, working together with health personnel in the village for the delivery of the LF drugs. Within the Indonesian context, cadres play an important role in the health of the village. Each village in Indonesia will have several cadres who are responsible for maternal and child health activities as well as any additional health activities planned in the village. They are renumerated with a small stipend for their activities.

Globally, research has shown the important role community drug deliverers (CDDs) play in the LF drug distribution [10]. As members of the community, they know how to reach people effectively and have been shown to improve community compliance with taking the LF drugs. In Indonesia they are specifically responsible for disseminating information about LF before MDA, drug delivery and reporting activities related to MDA. They work closely with the local health staff from the primary health care center (called *Puskesmas* in Indonesia) who are responsible for their training and supervision.

Despite all the efforts and gains that Indonesia has made in the LF elimination programme, achieving consistent and sufficient coverage (drug receipt) and compliance (drug ingestion) remains a challenge across many of the provinces in Indonesia [11]. This is a major concern as the 2020 deadline approaches for LF elimination [1]. One reason lies in the coverage - compliance gap that occurs in situations where directly observed treatment (DOT) is not implemented, meaning that drugs that are distributed are recorded as consumed, when actual consumption has not been confirmed by the distributor [12]. A complementary study to this one highlighted a coverage-compliance gap in the data reported in two of the study districts discussed in this paper [13]. The district health teams expected that all drugs distributed were consumed, however follow up research showed that only a proportion of recipients actually consumed the distributed LF drugs [13]. According to the international guidelines, LF drugs should be consumed using DOT [14]. In the Indonesian context, this would mean taking the pills in front of the cadres or the frontline health personnel*.* As the Indonesian program accelerates towards elimination, ensuring DOT will be important to ensuring effective MDA, i.e. that distributed drugs are consumed drugs.

In 2015, research was carried out in Indonesia by the Center for Health Research in the Faculty of Public Health at Universitas Indonesia in partnership with researchers from the USA, Canada and Australia, to assess coverage and compliance in three districts and to increase understanding as to why drug coverage may be persistently low, what specific actions may be undertaken to improve delivery and uptake and how those responsible for delivering MDA may be better supported. The research described here complements a community-based study using micro narratives [13] and was performed in the same catchment areas, i.e. the City of Depok (West Java Province), the City of Batam (Kepulauan Riau Province) and Agam District (West Sumatera Province). The research tool used here also included micro narratives; however, they are not presented here in the analysis. Using data from this research, this paper aims to assess knowledge about LF and the implementation of MDA for LF amongst drug deliverers in those three districts/cities of Indonesia. Understanding how drug distributors participate in MDA, know the eligibility criteria and retain basic scientific knowledge will be important so that elimination programs know how to better support improvements to program reach, particularly in areas of low coverage.

## Methods

### Data source and study sites

**Fig. 1:**
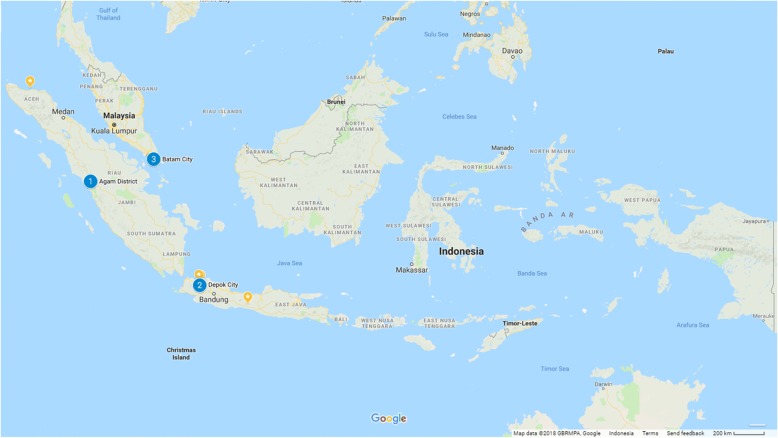
Map of study sites

The study used information from a cross-sectional survey conducted from January to March 2015 in one district and two cities of Indonesia: Agam District (West Sumatera Province), the City of Depok (West Java Province) and the City of Batam (Kepulauan Riau Province) (Fig. 1). Based on the recommendation from the national LF Elimination Programme, Ministry of Health, Republic of Indonesia, these three areas were proposed due to persistent challenges in the implementation of the previous MDA rounds. In 2015 when the surveys were implemented, Agam District and the City of Depok had been actively working to improve their MDA since baseline surveys in 2014. This study reflects the baseline results for the City of Batam.

### Background information of study sites

Agam District, located on the western coast of West Sumatera Province, has a population of just over 450,000 based on the 2010 Population Census [15] which was distributed over 2300 km^2^ (190 people per km^2^). The primary economic activity is agriculture. The LF species in this area is *Brugia malayi* [11]. In 2014, the microfilaria (mf) prevalence in Agam District ranged between 7.3–8.7% [16]. Mass drug administration began in 2005 with partial geographical coverage. In 2007, the district was implementing MDA across the whole district. At the time of this research (2015), the District Health Authority in Agam had conducted five rounds across the whole district (i.e. full geographical coverage). According to information from District Health Office of Agam, drug coverage ranged between 89.6–96.7% in 2015.

The City of Depok is part of the greater metropolitan area of Indonesia known as *Jabodetabek* (Jakarta, Bogor, Depok, Tangerang and Bekasi)*.* Depok is located in West Java Province, with the population of 1.75 million based on the 2010 Population Census [17] in an area of 200 km^2^ (8746 people per km^2^). In 2007, the mf prevalence was measured at 2.8% in a population of 500 people. In 2012, the mf prevalence in Depok was 2.01% [18]. The LF species in this area is *Wuchereria bancrofti.* The City of Depok began MDA in 2006 with partial geographical coverage. By the time this research began in 2013, the City of Depok had completed five rounds of MDA at full geographical coverage, with coverage varying between 46–84%, as per district records.

The City of Batam is the largest city in Riau Islands Province, located across the strait of Singapore. The total population of Batam based on the 2010 Population Census was 944,285 people, with population density of 940 people/km^2^. As an industrial city, Batam is included in the free-trade zone Indonesia-Malaysia-Singapore Growth Triangle. The LF species in this area is *Brugia malayi*. According to information from the Health Office of Batam City, by 2016, Batam had conducted MDA five times since 2013 and the coverage during this time were between 51.8–73.1%, per district records.

### Study design and samples of the study

In each district the research team held consultations with LF program managers in the Health Office to determine the best areas for the research. Some sub-districts were identified as having more challenges related to MDA coverage than others and as such, they were selected purposively for the research. The sub-districts selected were: (i) in Agam District: Ampek Nagari, Tanjung Mutiara and Lubuk Basung; (ii) in the City of Depok: Tapos, Limo, Bojong Sari, Sukmajaya, Beji; and (iii) in the City of Batam: Bengkong, Galang, Sagulung.

In each district, two research components were carried out: a community survey, whose methods and results are reported in Krentel et al. [13] and a survey administered to those involved with drug delivery living in the same sub-districts as the community survey. This paper reports on the methods and quantitative data from the deliverer survey. Because health personnel and community leaders also participate in the MDA together with the cadrers, it was decided that all three groups should be included in the sample as “drug deliverers”.

In each district, 100 respondents were selected using purposive sampling from the hamlet, village and sub-district level. The respondents had to have been involved in the last MDA activity (either in promotion or implementation), and were identified as cadres from the hamlet level: village midwives; community or religious leaders (village level); the head of health center; the local LF program manager; and community or religious leaders (sub-district level).

### Survey instruments and field personnel

A questionnaire was designed to allow for broad examination of factors associated with the MDA process, namely factors associated with facilitating effective drug delivery (e.g. training, supervision, incentives, obligation to superiors); perception of community engagement with the MDA process; opinions about the LF drugs (e.g. size, taste, packaging, safety); and respondent’s role in the MDA (e.g. tasks, perception of self-confidence to carry out their tasks, perceived importance of their role).

A fieldwork team was established in each district led by one district field coordinator (DFC) with two local interviewers recruited from each study site. DFCs and enumerators attended a two-day training program followed by a one-day supervised practice in the field. The training sought to familiarise respondents with the survey methodology, the questionnaires and interview techniques. Following the training, local enumerators implemented the survey. At the end of each day of data collection, the enumerators, field coordinators and members of the research team reviewed the questionnaires and checked them for any issues.

The questionnaire had certain similarities with the community survey conducted concurrently, namely that it used micronarratives as the basis for better understanding of the deliverers’ experience. This methodology has been described elsewhere [13, 19]. The question used to elicit the deliverer’s experience was, “tell me about one experience that was your most memorable when you gave the drugs out or when you were raising awareness about MDA”. Following that micronarrative, participants were asked to answer a series of closed explanatory questions that provided additional insight into the respondent’s experience. Respondents were also asked a series of questions eliciting their knowledge of three aspects of LF and LF delivery (see below for details). This paper presents the analysis of the explanatory and knowledge questions.

### Outcome variables

In this analysis, we used three outcome variables: (i) knowledge about LF; (ii) knowledge about MDA implementation; and (iii) a proxy indicator for feedback in MDA based on being informed about the number of people taking LF drugs (MDA feedback). The scores assigned to variables used to construct each outcome were summed to provide a total score for each outcome for each respondent. For the purpose of this analysis all questions were considered to be of equal weight.

For the oucome variable of “knowledge about LF”, three variables were used: (i) know that worm is the cause of LF; (ii) know that mosquitoes transmit LF; and (iii) know that LF is preventable. A score of 1 was assigned where a question was answered correctly and 0 otherwise. After identifying the median distribution of the scores, we categorized individuals scored less than median as having a “low level of knowledge”; whereas individuals scored the same as median or above as having a “high level of knowledge” about LF for the purposes of this research.

For the outcome of “knowledge about MDA”, six variables were used: knowledge that (i) all LF drugs should be taken; (ii) pregnant women should not take LF drugs; (iii) children under two years old should not take LF drugs; (iv) severely undernourished children should not take LF drugs; (v) people aged more than 75 years-old should not take LF drugs; and (vi) severely ill people should not take LF drugs. A score of 1 was assigned to each question answered correctly and 0 if otherwise. The total score for this outcome thus ranged from 0 to 6. As with the previous score, using median distribution of respondents’ scores as the cut-off point, individuals scored less than median was considered as having a “low level of knowledge” and individuals scored the same as median or above as having a “high level of knowledge” about MDA for the purposes of this research.

For the outcome variable of “MDA feedback”, the variable was based on one question about whether respondent was informed about the number of people who received or took LF drugs. Score 1 was assigned to respondent who answered “yes” and score 0 otherwise. This score represented the feedback received by the deliverer about the outcome of the last MDA.

### Potential predictors

Predictor variables were used to explain or predict the value of outcome variables. Three groups of predictors were used: (i) socio-demographic characteristics: district/city (Agam, Depok, Batam); age of respondents (≤ 35 years, 36–45 years and ≥ 46 years); sex (male and female); highest level of educational attainment (no school/incomplete primary/completed primary school); length of stay in the area (≤ 2 years, > 2 years); (ii) role and process of delivering LF drugs: role of respondents during MDA (cadre, community/religious leaders, health workers); experience of working with others during MDA (yes and no); and (iii) knowledge and training to perform in MDA: perceived adequacy of knowledge to carry out roles and responsibilities in MDA (inadequate, neutral and adequate); and perception about training prior to MDA (very informative, informative, less informative, nothing).

### Data analysis

We examined the characteristics of all variables (outcome variables and potential predictors) using contingency tables. Logistic regression analyses were used to determine factors associated with all outcome variables. The estimated measures of association were assessed using ORs (odds ratios). In the first stage of logistic regression analysis, bivariate regression analyses were employed to assess the relationship between outcome variables and their potential predictors, independently. Afterwards, multivariate analyses were performed to examine the association between outcome variables and potential predictors. We used backward elimination method to remove all variables not significantly related to the study outcome using the significance level of 0.05. The variables district, age and role of respondent during MDA were selected a priori and were retained in the final model regardless of the significance level. In the final model, adjusted ORs (aOR) and 95% confidence intervals (95% CIs) were determined for all variables in the model. All estimates presented in this analysis considered the complex sample design. We used Stata/MP software (version 13.1; StataCorp) for all analyses. The results of the research have been presented to the District Health Office in all three districts as well as the National LF Programme, for their consideration and input into the analysis in a half-day workshop in Jakarta.

## Results

In this study we interviewed 318 people who had been involved in delivering LF drugs during the last MDA: 109 from Agam District, 107 from Depok and 102 from Batam. The distribution of respondents by socio-demographic characteristics as well as their role and intergration with other programs when delivering LF drugs during MDA is shown in Table 1. Around 75% of respondents completed at least senior high school, 95% stayed in the study area for more than two years, and more than half of the respondents were cadres. When respondents were asked about their experience in the last MDA, the majority reported that almost all community members were aware of the MDA. Most respondents reported MDA was conducted as an individual health activity and was not integrated with other programs. Only 20% of respondents reported integration with other programs during MDA, the highest in Depok (29%), followed by Agam (22.9%) and Batam (8.8%).

**Table 1:** Frequency distribution of drugs deliverers in MDA according to their socio-demographic charateristics, role in MDA and outcome variables

Variable	Frequency (total)			High knowledge about LF^a^			High knowledge about MDA^b^			Received MDA feedback		
	*n*	%	95% CI	*n*	%	95% CI	*n*	%	95% CI	*n*	%	95% CI
A. Socio-demographic characteristics												
District/City												
Agam	109	34.3	29.2–39.7	55	50.5	41.1–59.8	54	49.5	40.2–58.9	53	48.6	39.3–58.0
Depok	107	33.7	28.6–39.1	49	45.8	36.5–55.4	49	45.8	36.5–55.4	85	79.4	70.7–86.1
Batam	102	32.1	27.2–37.4	26	25.5	17.9–34.9	31	30.4	22.2–40.1	70	68.6	58.9–76.9
Age												
≤ 35 years	66	20.8	16.6–25.6	28	42.4	31.0–54.7	37	56.1	43.8–67.6	40	60.6	48.3–71.7
36–45 years	138	43.4	38.0–48.9	61	44.2	36.1–52.6	62	44.9	36.8–53.4	93	67.4	59.1–74.7
≥ 46 years	114	35.9	30.7–41.3	41	36.0	27.6–45.2	35	30.7	22.9–39.8	75	65.8	56.6–74.0
Sex												
Male	65	20.4	16.3–25.3	18	27.7	18.1–39.9	19	29.2	19.4–41.5	31	47.7	35.8–59.9
Female	253	79.6	74.7–83.7	112	44.3	38.2–50.5	115	45.5	39.4–51.7	177	70.0	64.0–75.3
Education group												
No school/incomplete primary school/completed primary school	25	7.9	5.4–11.4	5	20.0	8.4–40.6	9	36.0	19.6–56.5	11	44.0	25.9–63.8
Completed secondary	51	16.0	12.4–20.5	17	33.3	21.7–47.4	21	41.2	28.5–55.2	33	64.7	50.6–76.7
Completed senior HS	147	46.2	40.8–51.8	56	38.1	30.6–46.3	55	37.4	29.9–45.6	93	63.3	55.1–70.7
Completed college/above	95	29.9	25.1–35.2	52	54.7	44.6–64.5	49	51.6	41.5–61.5	71	74.7	65.0–82.5
Length of stay in the area												
≤ 2 years	15	4.7	2.9–7.7	9	60.0	33.9–81.4	6	40.0	18.6–66.1	11	73.3	45.6–90.0
> 2 years	302	95.0	91.9–96.9	120	39.7	34.3–45.4	127	42.1	36.6–47.7	196	64.9	59.3–70.1
B. Experience, role and integration with other programs during MDA												
Role of respondents during MDA												
Cadre	175	55.0	49.5–60.5	66	37.7	30.8–45.2	72	41.1	34.1–48.6	112	64.0	56.6–70.8
Community/religious leaders	84	26.4	21.8–31.6	20	23.8	15.8–34.2	23	27.4	18.9–38.0	43	51.2	40.5–61.8
Health workers (village midwives, head of health centre, LF program manager)	59	18.6	14.6–23.2	44	74.6	61.8–84.2	39	66.1	53.1–77.1	53	89.8	79.0–95.4
Frequency of participation in MDA												
1–3 times	170	53.5	47.9–58.9	49	28.8	22.5–36.1	58	34.1	27.3–41.6	101	59.4	51.8–66.6
> 3 times	148	46.5	41.1–52.1	81	54.7	46.6–62.6	76	51.4	43.3–59.4	107	72.3	64.5–79.0
Integration with other program during MDA												
No	253	79.6	74.7–83.7	91	36.0	30.3–42.1	99	39.1	33.3–45.3	159	62.8	56.7–68.6
Yes	65	20.4	16.3–25.3	39	60.0	47.6–71.3	35	53.8	41.6–65.6	49	75.4	63.4–84.4

^a^Knowledge about LF is based on three variables: (i) know that worm is the cause of LF; (ii) know that mosquitoes transmit LF; and (iii) know that LF is preventable. Low level of knowledge is assigned to those scoring less than median of the distribution and high level of knowledge is assigned to those scoring the same as median or above

^b^Knowledge about MDA is based on six variables: knowledge that (i) all LF drugs should be taken; (ii) pregnant women should not take LF drugs; (iii) children under two years old should not take LF drugs; (iv) severely undernourished children should not take LF drugs; (v) people aged more than 75 years old should not take LF drugs; and (vi) severely ill people should not take LF drugs. Low level of knowledge is assigned to those scoring less than median of the distribution and high level of knowledge is assigned to those scoring the same as median or above

### Frequency distribution of outcome variables by different characteristics

**Fig. 2:**
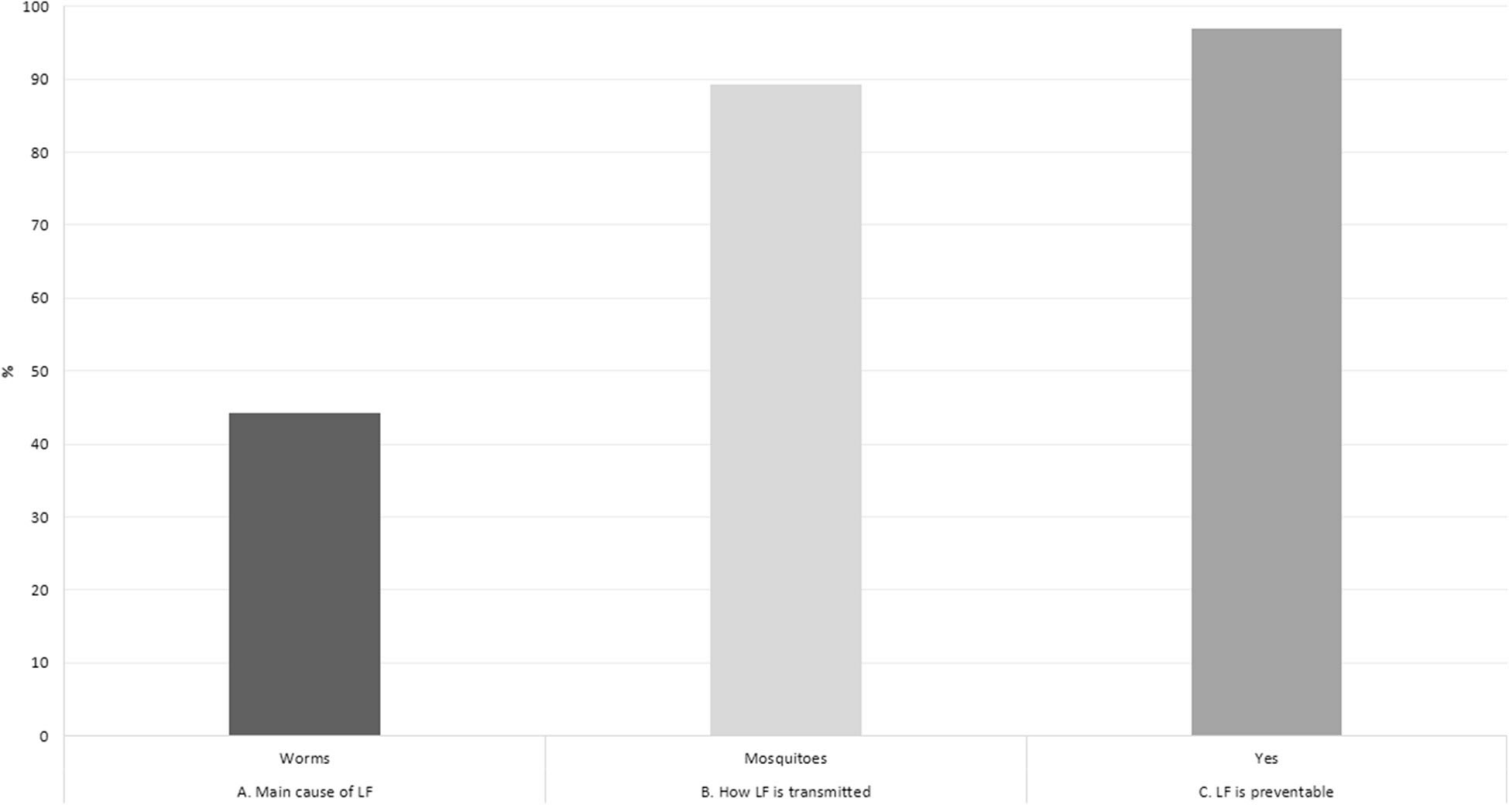
Knowledge of lymphatic filariasis

Across the three sites, 50% or less of respondents interviewed in this study had a high level of knowledge about LF (Table 1), particularly in the City of Batam, where only 25.5% of respondents had a high level of knowledge about LF. Figure 2 shows than less than 50% of respondents were aware that worms were the primary cause of LF. However, almost 90% of respondents stated that LF is preventable.

**Fig. 3:**
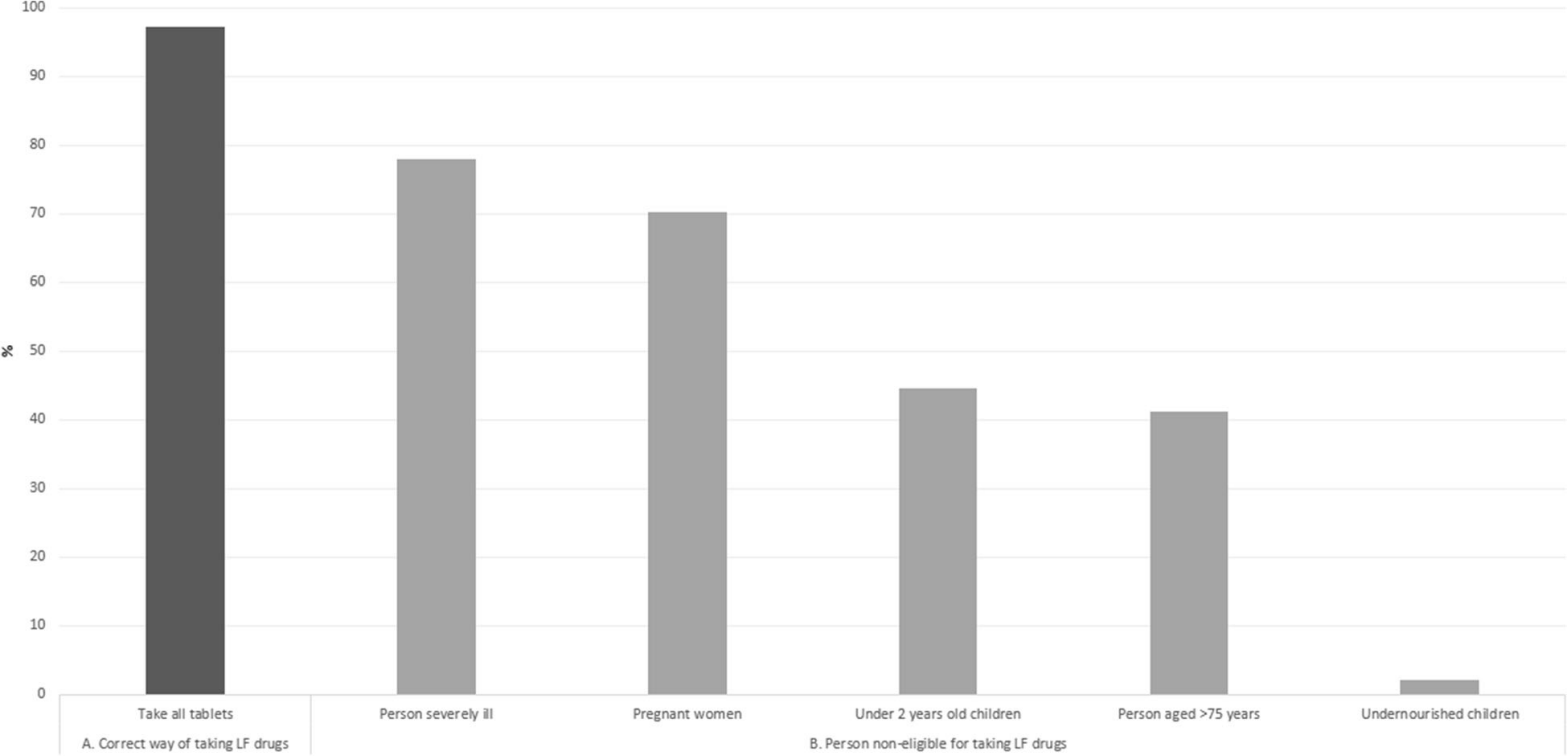
Knowledge about MDA

Similarly, less than half of respondents were assessed as having a high level of knowledge about MDA (Table 1). Less than half of respondents were aware that children under two years old, person aged more than 75 years and undernourished children were not eligible to take LF drugs (Fig. 3). Nevertheless, more than 75% of LF drugs deliverers stated that their knowledge was already adequate to help them conduct their work. A majority of deliverers surveyed also perceived that the information they received was sufficient to help them perform their tasks and responsibilities during MDA (Table 2).

**Table 2:** Frequency distribution of drugs deliverers in MDA according to their knowledge about LF and MDA as well as MDA feedback by their role during MDA

Variable	Frequency (total) role during MDA			Cadre (*n* = 175)			Religious/community leader (*n* = 84)			Health workers (*n* = 59)		
	*n*	%	95% CI	*n*	%	95% CI	*n*	%	95% CI	*n*	%	95% CI
A. Knowledge about lymphatic filariasis												
Knowledge of LF^a^												
Low	188	59.1	53.6–64.4	109	62.3	54.8–69.2	64	76.2	65.8–84.2	15	25.4	15.9–38.2
High	130	40.9	35.6–46.4	66	37.7	30.8–45.2	20	23.8	15.8–34.2	44	74.6	61.8–84.2
B. Knowledge about MDA												
Knowledge about MDA^b^												
Low	183	57.6	52.0–62.9	102	58.3	50.8–65.4	61	72.6	62.0–81.2	20	33.9	22.9–47.0
High	134	42.1	36.8–47.7	72	41.1	34.1–48.6	23	27.4	18.9–38.0	39	66.1	53.1–77.1
C. MDA feedback												
Informed about the number of people receiving or taking LF drugs												
No	110	34.6	29.5–40.0	63	36.0	29.2–43.4	41	48.8	38.2–59.5	6	10.2	4.6–21.0
Yes	208	65.4	60.0–70.5	112	64.0	56.6–70.8	43	51.2	40.5–61.8	53	89.8	79.0–95.4
D. Other												
Perceived adequacy of knowledge to carry out roles and responsibilities in MDA												
Inadequate	28	8.8	6.1–12.5	13	7.4	4.4–12.4	11	13.1	7.4–22.2	4	6.8	2.5–16.9
Neutral	50	15.7	12.1–20.2	27	15.4	10.8–21.6	15	17.9	11.0–27.6	8	13.6	6.9–25.0
Perception about training received prior to MDA												
Very informative	117	36.8	31.6–42.3	56	32.0	25.5–39.3	31	36.9	27.2–47.8	30	50.9	38.2–63.4
Informative	152	47.8	42.3–53.3	94	53.7	46.3–61.0	37	44.1	33.8–54.9	21	35.6	24.4–48.7
Less informative	29	9.1	6.4–12.8	14	8.0	4.8–13.1	10	11.9	6.5–20.8	5	8.5	3.5–19.0
No training	20	6.3	4.1–9.6	11	6.3	3.5–11.0	6	7.1	3.2–15.1	3	5.1	1.6–14.8

^a^Knowledge about LF is based on three variables: (i) know that worm is the cause of LF; (ii) know that mosquitoes transmit LF; and (iii) know that LF is preventable. Low level of knowledge is assigned to those scoring less than median of the distribution and high level of knowledge is assigned to those scoring the same as median or above

^b^Knowledge about MDA is based on six variables: knowledge that (i) all LF drugs should be taken; (ii) pregnant women should not take LF drugs; (iii) children under two years old should not take LF drugs; (iv) severely undernourished children should not take LF drugs; (v) people aged more than 75 years old should not take LF drugs; and (vi) severely ill people should not take LF drugs. Low level of knowledge is assigned to those scoring less than median of the distribution and high level of knowledge is assigned to those scoring the same as median or above

**Fig. 4:**
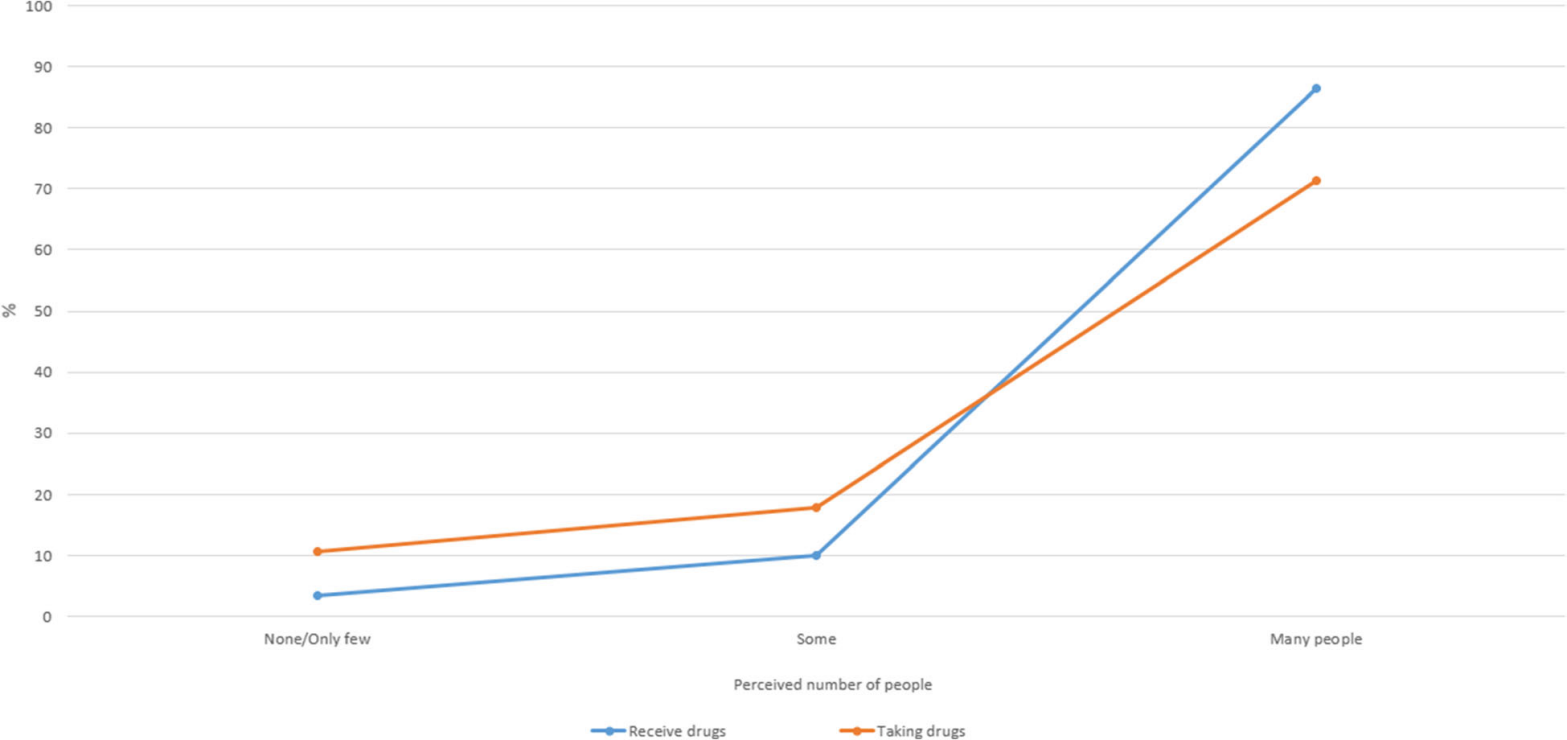
Perceived number of people receiving and taking LF drugs

On average, 65% of drugs deliverers across all study sites claimed that they had been informed about the number of people who received or swallowed the LF drugs at the end of the last MDA (MDA feedback) (Table 1). About 86% of respondents perceived that many people had received the LF drugs in the last MDA; however when asked about their perception of those who had swallowed the pills, only 71% reported that “many people had taken them” (Fig. 4).

**Fig. 5:**
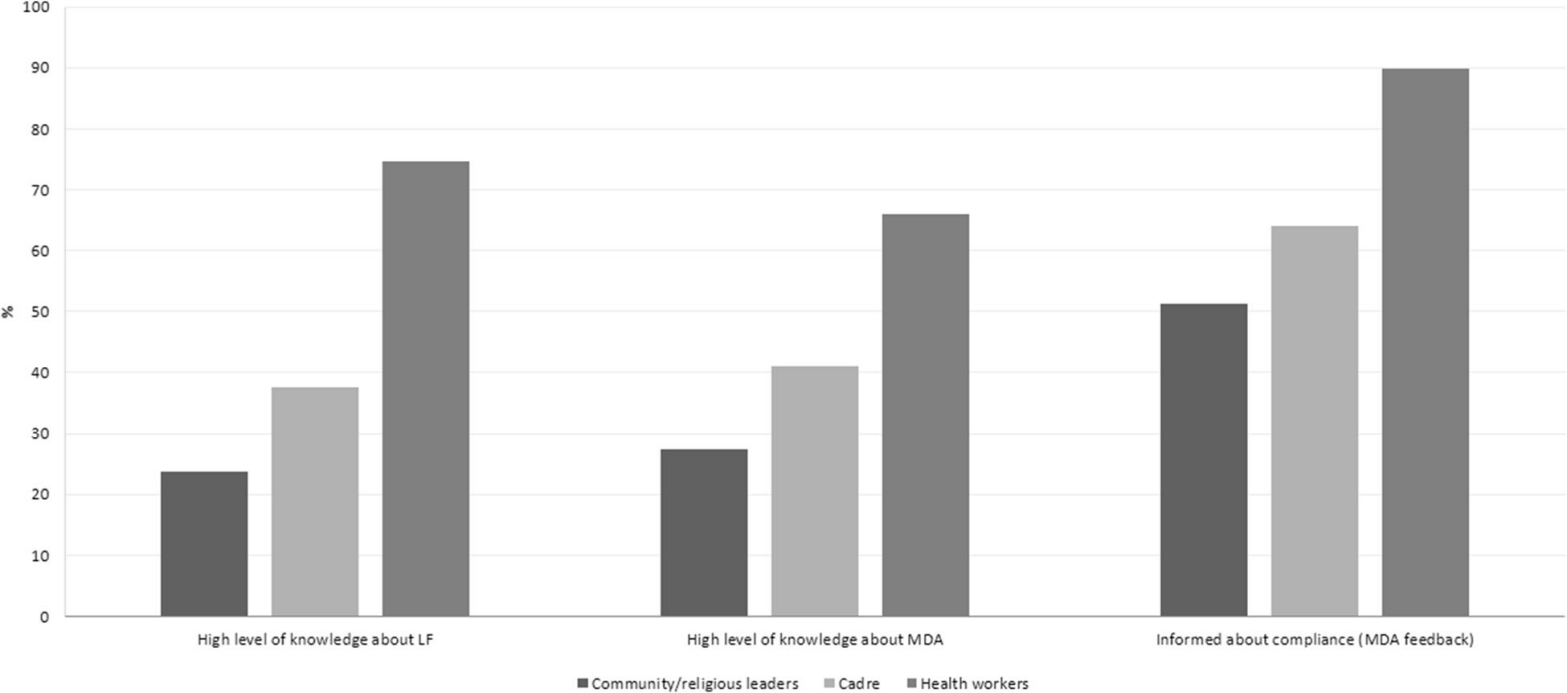
Level of knowledge about LF and MDA, and MDA feedback by type of drugs deliverers

By role during MDA, the distribution of respondents differed between the three outcome variables as shown in Table 2. The highest proportion of deliverers who had a high level of knowledge of LF, MDA and feedback about MDA were health workers (i.e. village midwives, head of health center, LF program manager), and followed by cadres (Fig. 5). The proportion of health workers with a high level knowledge about MDA was still below 70%, whereas the proportion of cadres and community leaders who had a high level of knowledge about LF and MDA were all below 45% (Fig. 5).

By study sites, the proportion of respondents who perceived their knowledge was adequate to help them in carrying out their roles and responsibilities during the MDA was the highest in Agam District (83.5%) and the lowest in the City of Batam (66.7%) (Table 3). The City of Batam also had the lowest proportion of LF drugs deliverers who had a high level of knowledge about LF and MDA amongst the three sites.

**Table 3:** Frequency distribution of drugs deliverers in MDA according to their knowledge about LF and MDA as well as MDA feedback by district

Variable	District								
	Agam District			Depok City			Batam City		
	(*N* = 109)			(*N* = 107)			(*N* = 102)		
	*n*	%	95% CI	*n*	%	95% CI	*n*	%	95% CI
A. Knowledge about lymphatic filariasis (LF)									
Knowledge of LF^a^									
Low	54	49.5	40.2–58.9	58	54.2	44.7–63.5	76	74.5	65.1–82.1
High	55	50.5	41.1–59.8	49	45.8	36.5–55.4	26	25.5	17.9–34.9
B. Knowledge about MDA									
Knowledge about MDA^b^									
Low	54	49.5	40.2–58.9	58	54.2	44.7–63.5	71	69.6	59.9–77.8
High	54	49.5	40.2–58.9	49	45.8	36.5–55.4	31	30.4	22.2–40.1
C. MDA feedback									
Informed about the number receiving or taking LF drugs									
No	56	51.4	42.0–60.7	22	20.6	13.9–29.3	32	31.4	23.1–41.1
Yes	53	48.6	39.3–58.0	85	79.4	70.7–86.1	70	68.6	58.9–76.9
D. Other									
Perceived adequacy of knowledge to carry out roles and responsibilities in MDA									
Inadequate	10	9.2	5.0–16.3	9	8.4	4.4–15.5	9	8.8	4.6–16.2
Neutral	8	7.4	3.7–14.1	17	15.9	10.1–24.2	25	24.5	17.1–33.9
Adequate	91	83.5	75.2–89.4	81	75.7	66.6–83.0	68	66.7	56.9–75.2
Perception about training received prior to MDA									
Very informative	22	20.2	13.6–28.8	59	55.1	45.6–64.4	36	35.3	26.6–45.1
Informative	68	62.4	52.9–71.0	38	35.5	27.0–45.1	46	45.1	35.7–54.9
Less informative	7	6.4	3.1–12.9	4	3.7	1.4–9.6	18	17.7	11.4–26.4
No training	12	11.0	6.3–18.5	6	5.6	2.5–12.0	2	2.0	0.5–7.6

^a^Knowledge about LF is based on three variables: (i) know that worm is the cause of LF; (ii) know that mosquitoes transmit LF; and (iii) know that LF is preventable. Low level of knowledge is assigned to those scoring less than median of the distribution and high level of knowledge is assigned to those scoring the same as median or above

^b^Knowledge about MDA is based on six variables: knowledge that (i) all LF drugs should be taken; (ii) pregnant women should not take LF drugs; (iii) children under two years old should not take LF drugs; (iv) severely undernourished children should not take LF drugs; (v) people aged more than 75 years old should not take LF drugs; and (vi) severely ill people should not take LF drugs. Low level of knowledge is assigned to those scoring less than median and high level of knowledge is assigned to those scoring the same as median or above

### Multivariable analysis of the three outcome variables

Table 4 shows the results of the multivariable analyses aimed to examine factors associated with three outcome variables used in this study. For the first indicator set, the odds of having a high level of knowledge about LF was lower in the City of Batam (aOR = 0.39, 95% CI: 0.– 0.85, *P* = 0.017) than in Agam District. As may be expected, the odds of having a high level of knowledge about LF amongst health workers were more than six times the odds of cadres (aOR = 6.47, 95% CI: 3.03–13.83, *P* < 0.001). Amongst deliverers reporting that there was no training, the odds of having high level of knowledge about LF was five times lower than those who considered the training to be highly informative (aOR = 0.21, 95% CI: 0.05–0.84, *P* = 0.027).

**Table 4:** Multivariate analysis on factors associated with knowledge about LF and MDA and MDA feedback

Variable	High level of knowledge about LF^a^				High level of Knowledge about MDA^b^				Received MDA feedback^c^			
	aOR	95% CI		*P*-value	aOR	95% CI		*P*-value	aOR	95% CI		*P*-value
A. Socio demographic characteristics												
District/City												
Agam District	1.00				1.00				1.00			
Depok City	0.89	0.46–1.70		0.720	1.03	0.57–1.86		0.922	5.08	2.53–10.17		< 0.001
Batam City	0.39	0.18–0.85		0.017	0.62	0.31–1.24		0.175	2.54	1.33–4.86		0.005
Age												
≤ 35	1.00				1.00				1.00			
36–45	1.46	0.71–2.98		0.301	0.71	0.37–1.37		0.309	1.77	0.86–3.67		0.123
46–55	0.83	0.39–1.79		0.643	0.31	0.15–0.64		0.002	1.92	0.87–4.25		0.107
Sex												
Male									1.00			
Female									2.68	1.07–6.75		0.036
B. Role and frequency of participation in MDA												
Role of respondent during MDA												
Cadre	1.00				1.00				1.00			
Community/religious leaders	0.57	0.30–1.08		0.086	0.66	0.36–1.21		0.178	0.95	0.41–2.21		0.904
Health workers	6.47	3.03–13.83		<0.001	2.68	1.40–5.14		0.003	9.01	3.32–24.44		< 0.001
Frequency of participation in MDA												
1–3 times	1.00				1.00							
> 3 times	2.42	1.26–4.71		0.008	2.10	1.12–3.92		0.021				
C. Knowledge and training to perform in MDA												
Perceived adequacy of knowledge to conduct their tasks and responsibility in MDA												
Inadequate									1.00			
Neutral									8.26	2.55–26.74		< 0.001
Adequate									6.72	2.48–18.22		< 0.001
Perception about training prior to MDA												
Very informative	1.00											
Informative	1.31	0.73–2.36		0.371								
Less informative	1.43	0.52–3.97		0.491								
No training	0.21	0.05–0.84		0.027								

^a^Other variables included in the analysis but were removed in multivariate analysis: sex, education, length of stay, work with other during MDA, perceived adequacy of knowledge to carry out roles and responsibilities in MDA. Knowledge about LF is based on three variables: (i) know that worm is the cause of LF; (ii) know that mosquitoes transmit LF; and (iii) know that LF is preventable. Low level of knowledge is assigned to those scoring less than median of the distribution and high level of knowledge is assigned to those scoring the same as median or above

^b^Other variables included in the analysis but were removed in multivariate analysis: sex, education, length of stay, work with other during MDA, perceived adequacy of knowledge to carry out roles and responsibilities in MDA, perception about training received prior to MDA. Knowledge about MDA is based on six variables: knowledge that (i) all LF drugs should be taken; (ii) pregnant women should not take LF drugs; (iii) children under two years old should not take LF drugs; (iv) severely undernourished children should not take LF drugs; (v) people aged more than 75 years old should not take LF drugs; and (vi) severely ill people should not take LF drugs. Low level of knowledge is assigned to those scoring less than median of the distribution and high level of knowledge is assigned to those scoring the same as median or above

^c^Other variables included in the analysis but were removed in multivariate analysis: education; length of stay; work with other during MDA; perception about training received prior to MDA; knowledge of LF; and knowledge about MDA, and frequency of participation in MDA *Abbreviation*: *aOR* Adjusted odds ratio

Similarly, for the second outcome variable, the odds of having a high level of knowledge about MDA amongst drugs deliverers aged 46 years and above were about 70% the odds of those aged 35 years or less (aOR: 0.31, 95% CI: 0.15–0.64, *P* = 0.002). The odds of having a high level of knowledge about MDA was more than twice as high in health workers relative to cadres (aOR: 2.68, 95% CI: 1.40–5.14, *P* = 0.003) (Table 4).

For the third outcome variable, our analysis showed that LF drugs deliverers in the City of Batam were more likely to be informed (receive MDA feedback) about the number of people taking LF drugs than in Agam District (aOR: 2.54, 95% CI: 1.33–4.86, *P* = 0.005) (Table 4). Female deliverers were also more likely to receive feedback than male (aOR: 2.68, 95% CI: 1.07–6.75, *P* = 0.036). As expected, health workers were nine times more likely to receive MDA feedback than cadres (aOR: 9.01, 95% CI: 3.32–24.44, *P* < 0.001). Drugs deliverers who perceived that their knowledge to perform their tasks and responsibility in MDA was adequate, were also more likely to receive feedback about community’s compliance in MDA (aOR: 6.72, 95% CI: 2.48–18.22, *P* < 0.001) than those perceiving that their knowledge to perform their tasks and responsibility in MDA was inadequate.

## Discussion

Our understanding of the knowledge of community drug deliverers have about LF and MDA is not well researched globaly. This paper represents one of a few studies with an aims to assess the knowledge, perceptions and experiences of drug deliverers within an LF elimination campaign. Most of the peer-reviewed literature exploring issues relating to deliverers has been predominantly based within the African context [20]. While some of the same issues will apply to deliverers in the Asian context, others will not. For example early research established differences in preferences for MDA delivery in India and Ghana, notably the involvement of frontline health personnel in India *versus* community volunteers in Ghana [21, 22]. The LF programme in Indonesia exhibits certain characteristics: (i) it is a single disease programme, rather than an integrated NTD programme; (ii) drug delivery for LF is carried out together by health personnel and cadres rather than largely by volunteer drug distributors; (iii) mass drug administration for LF is not integrated with any other NTD activities. Note that other NTDs present in Indonesia include: schistosomiasis, leprosy, yaws, scabies and soil-transmitted helminths. The high burden of soil-transmitted helminths (STH) in some parts of Indonesia warrants inclusion of promoting treatment of STH during MDA for LF [23].

This research provides some insights into the knowledge of deliverers working in the Indonesian LF programme by exploring three knowledge attributes of those involved in the delivery of the LF drugs during MDA, specifically related to: (i) lymphatic filariasis; (ii) the mass drug administration; and (iii) coverage and compliance results (MDA feedback) within the deliverer’s location. Each of these three areas will be discussed within the context of the broader literature.

There were varied levels of knowledge about LF amongst the three different groups of deliverers approached: community leaders, cadres and health workers. Overall, health workers have a higher level of knowledge than cadres, which may be expected. Community and religious leaders however have much less knowledge than cadres. These individuals may not be routinely included in the regular cadre training and may not even receive formal training. They are, however, important spokespeople for the programme within the community and play a crucial role in mobilising the community and encouraging people to take the LF pills. When drug deliverers and community promoters are not well informed, there is a risk that incorrect information may be transmitted, conflicting with information from the health services. This discrepancy risks to undermine community confidence in both the deliverer and the MDA process itself. We know from other contexts that community confidence is important to encourage compliance with the treatment. In Sri Lanka, Gunawardena et al. [24] reported that people were unhappy to take the LF drugs from the distributors when there was inadequate information. Weerasooriya et al. [25] remarked that people did not have confidence in some of the distributors, thus effecting compliance in the community under study. In India, Ramaiah at al. [22, 26] showed that the community had poor confidence in the distributors. Our study shows that those who have more experience with MDA (> 3 times) have higher levels of knowledge than those who have participated less than 3 times. This suggests that repeated exposure to training may impact knowledge and understanding. Training that is tailored to the level of experience of the deliverer is recommended so that those participating in MDA for the first few rounds might receive more detailed instruction and supervision than those who have been part of the MDA programme for years.

Of our three research sites, Batam City consistently scored lower on LF knowledge and perception of training in relation to the other sites. We posit that there are several reasons for this difference: the urban nature of Batam City and the challenges inherent in MDA programs in these areas; systemic issues within the local LF program that require further investigation; and the measurement of Depok and Agam deliverers was conducted after a year of enhanced MDA activity by the DHO. The other urban area in our study, Depok City, is part of the greater Jakarta metropolitan area, and so may benefit from higher exposure to the LF programme in terms of support and materials thereby differentiating it from Batam.

In our study, deliverers seem to recognise the coverage-compliance gap as seen in their perceptions of how many community members received drugs *versus* those who they perceived had swallowed the pills. Babu & Babu [12] used the term “coverage-compliance” gap to explain the inconsistency between the recorded distributed drugs and those drugs that are ingested. This reflects the common digression from the gold standard practice of directly observed treatment (DOT) whereby individuals are expected to swallow the LF drugs in front of the deliverer [27]. In Indonesia, over time, the practice of DOT is not routinely followed by drug deliverers. As a result, coverage results do not always reflect consumed drugs. Our study showed that the deliverers themselves perceive this gap between coverage and compliance as revealed in the discrepancy between their perception of drugs distributed and drugs consumed. Any improvements to the MDA program will need to address poor adherence of drug deliverers to the standard of DOT. To support this, the manner of distribution will need to be assessed (house to house *versus* distribution posts), the perception of community members on the need to take treatment before bed as well as the need to eat before swallowing pills and improved supervision during MDA. These factors can impede the direct observation of drug ingestion in front of drug deliverers as has been shown in Indian studies where DOT has not been ensured [28–30].

The study participants had a surprisingly low score for knowledge about mass drug administration, particularly when it concerned the eligibility of individuals. This may be the result of some of the Indonesian ELF program changes on eligibility over time, particularly with regards to breastfeeding women and elderly persons. Particularly concerning was the poor overall knowledge of the ineligibility of children under two years old and undernourished children. In the published literature, evidence shows that communities perceive when deliverer knowledge with regards to eligibility is inadequate. In India, community members reported that they lacked confidence in the distributors’ ability to assess eligibility for DEC during the MDA [31]; and in another Indian study [30] community members reported that distributors were confused and gave inadequate amounts of the LF drugs. In addition to the effect on community perceptions and confidence in the distributor, there are also concerns with regards to misallocated treatment to individuals who are ineligible for treatment. Our results show that the longer deliverers are involved with the MDA program, the more likely they are to have better understanding about eligibility for MDA. This suggests that programmes need to focus on retaining the delivery workforce so as to build and capitalise on their knowledge and experience.

While participants had a low knowledge scores for MDA, they perceived that their knowledge is sufficient for the implementation of their work. In Agam District, this was stronger than in the other two more urban areas. The process of mass drug administration lends itself more to a rural or semi-rural environment, such as Agam, where cadres are usually active in the population and known to their communities. The content of the training and the application of MDA by community volunteers may be more appropriate for these contexts, rather than the urban environments of Batam and Depok where cadres may not be known in the population, where the community is more diverse and where population movement is more common place. These factors have been explored in other research related to MDA within urban settings [32–34]. In short, these results suggest that the Agam deliverers feel more confident in their MDA activities as the training may be more appropriate to their semi-rural and rural contexts. Achieving sufficient drug coverage in urban MDA programs remains a key challenge to the global program and our study suggests that renewed training would be an area for intervention [35].

This research is not without its limitations. One of the challenges in assessing drug deliverers is establishing a reliable outcome measurement. Because performance-based measurements are not usually carried out for drug deliverers, any variable of success in activities will be subjective to the individual deliverer. If the deliverer reports success, can this be considered an accurate and real measurement? Furthermore, as the literature has stated [12], the coverage-compliance gap remains an important concern in the delivery of the LF drugs and as a result, these perceptions of success as measured in drugs delivered may not actually represent drugs consumed. So although our three outcomes of measurement, (i) knowledge about LF; (ii) knowledge about MDA; and (iii) MDA feedback, may not be the most appropriate measurements of performance of drug deliverers, they nevertheless can serve as proxy indicators for the training activities, supervision and evidence of the feedback loop in program monitoring and evaluation. Because purposive sampling was used to identify individuals, it is possible that some bias may have been introduced in the selection of individuals, although every effort was made to minimize any bias. The authors provided a matrix for each study site to ensure that a range of informants were identified. The study teams in each site followed this matrix closely.

## Conclusions

Our research demonstrates that there are variations in knowledge about LF and MDA as well as MDA feedback across the three different kinds of people involved with MDA delivery in Indonesia: the health personnel, cadres and community leaders. Although it is expected that there would be certain differences in between groups, these variations may also reflect differences in the training programmes prior to MDA as well as active supervision during the MDA process. Some of these variations are also seen in between the urban (Depok City and Batam) and the rural (Agam) sites of this research. The knowledge as well as level of confidence of the deliverer is different in the urban areas than in the rural location of the research, suggesting that the MDA platform may be more suitable for rural populations. Increased years of experience are related to higher levels of knowledge about LF and MDA suggesting that retaining the existing workforce is of paramount importance. Our research suggests some recommedations to improve the performance of drug deliverers. First, urgent action is required to solidify knowledge about eligibility across all groups working to promote and distribute LF drugs during MDA. Secondly, current guidelines for MDA can be adapted to an urban environment, in particular, updating deliverer training and promotional activities. Supportive supervision in these environments may help to motivate, increase confidence and build the skills of cadres in these locations. Thirdly, supporting materials and/or shortened training for community leaders, particularly in low performing areas will be beneficial to increase awareness and participation during MDA. Finally, regular assessments of the deliverer workforce is important to detect any fluctuations in knowledge amongst these individuals, due to turnover or lack of training. Training programmes need to reflect the level of experience and knowledge of deliverers, considering the different needs of those participating for the first time. Due to the long term nature of the LF elimination program, it is important that programs actively assess and aid these key individuals, retaining this key workforce.
